# Bioenergetic Health Assessment of a Single *Caenorhabditis elegans* from Postembryonic Development to Aging Stages via Monitoring Changes in the Oxygen Consumption Rate within a Microfluidic Device

**DOI:** 10.3390/s18082453

**Published:** 2018-07-28

**Authors:** Shih-Hao Huang, Yu-Wei Lin

**Affiliations:** Department of Mechanical and Mechatronic Engineering, National Taiwan Ocean University, Keelung 202-24, Taiwan; n490524@yahoo.com.tw

**Keywords:** *Caenorhabditis elegans*, oxygen consumption rates, bioenergetic health index

## Abstract

Monitoring dynamic changes in oxygen consumption rates (OCR) of a living organism in real time provide an indirect method of monitoring changes in mitochondrial function during development, aging, or malfunctioning processes. In this study, we developed a microfluidic device integrated with an optical detection system to measure the OCR of a single developing *Caenorhabditis elegans* (*C. elegans*) from postembryonic development to aging stages in real time via phase-based phosphorescence lifetime measurement. The device consists of two components: an acrylic microwell deposited with an oxygen-sensitive luminescent layer for oxygen (O_2_) measurement and a microfluidic module with a pneumatically driven acrylic lid to controllably seal the microwell. We successfully measured the basal respiration (basal OCR, in pmol O_2_/min/worm) of a single *C. elegans* inside a microwell from the stages of postembryonic development (larval stages) through adulthood to aged adult. Sequentially adding metabolic inhibitors to block bioenergetic pathways allowed us to measure the metabolic profiles of a single *C. elegans* at key growth and aging stages, determining the following fundamental parameters: basal OCR, adenosine triphosphate (ATP)-linked OCR, maximal OCR, reserve respiratory capacity, OCR due to proton leak, and non-mitochondrial OCR. The bioenergetic health index (BHI) was calculated from these fundamental parameters to assess the bioenergetic health of a single developing *C. elegans* from the postembryonic development to aging stages. The changes in BHI are correlated to *C. elegans* development stage, with the highest BHI = 27.5 for 4-day-old adults, which possess well-developed bioenergetic functionality. Our proposed platform demonstrates for the first time the feasibility of assessing the BHI of a single *C. elegans* from postembryonic development to aging stages inside a microfluidic device and provides the potential for a wide variety of biomedical applications that relate mitochondrial malfunction and diseases.

## 1. Introduction

The mitochondrion, an important cellular organelle, plays a central role in energy metabolism, where adenosine triphosphate (ATP), the energy currency of living cells, is produced via oxidative phosphorylation inside mitochondria. The mitochondrial ATP production relies on the electron transport chain (ETC), composed of respiratory chain complexes I–IV, which transfer electrons in a stepwise fashion until they finally reduce oxygen to form water, causing oxygen consumption [1]. It has been proven that the measurement of the oxygen consumption rate (OCR) of a living organism provides an indirect method to easily evaluate the energy metabolism of mitochondria [2,3,4,5,6]. Moreover, recent research has made it well known that development, aging, and longevity strongly correlate with the energy metabolism of mitochondria [7,8]. Monitoring dynamic changes in the OCR of a living organism in real time allows us to monitor changes in mitochondrial function as an index of these processes.

The nematode *Caenorhabditis elegans* (*C. elegans*), an important invertebrate model organism, offers many advantages over traditional mammalian models, including ease of maintenance, a short (2–3 weeks) lifespan, and fully established genetic information [9]. Particularly, *C. elegans* is a useful animal for assessing mitochondrial function in development and aging processes because its maximum lifespan is most closely related to energy metabolism. 

Recently, microfluidic devices have become a useful tool for *C. elegans* studies to solve major problems in the handling and manipulation of *C. elegans* via microfluidic techniques, with the advantages of high-throughput experimentation through parallelization and automation [10,11,12]. A variety of microfluidic devices have been developed to perform complicated operations, including the manipulation of *C. elegans* for precise spatial positioning to expose *C. elegans* to toxic agents or clinical drugs in a systematically controlled environment to evaluate the phenotypic changes in *C. elegans* [13,14,15,16]. However, few of the microfluidic platforms have been developed to quantitatively measure the mitochondrial function of *C. elegans* to understand the metabolic profiles of *C. elegans* during their lifecycle or to evaluate the physiological effects of testing agents on *C. elegans* at different life stages. Recently, a commercial microplate-based extracellular flux analyzer (Agilent Technologies Inc., Santa Clara, CA, USA) was used for OCR measurements to assay the mitochondrial bioenergetics in mutant *C. elegans* with mitochondrial dysfunction [5,6]. These measurements still require approximately 50 nematodes (*C. elegans*) to be pipetted into each well of 24-well microplates to have sufficient oxygen consumption for OCR measurements. The more nematodes the OCR measurements use, the greater the possibility that information about individual differences may be lost. Therefore, we attempted to trap a single *C. elegans* and develop a simple microfluidic device [17] that enabled us to continually measure the OCR with high sensitivity and reproducibility, to assess the mitochondrial bioenergetics of a single *C. elegans* from the larval stage to the aged adult stage. 

In this study, we developed a microfluidic device integrated with an optical detection system to measure the OCR of a single developing *C. elegans* via phase-based phosphorescence lifetime detection. The microfluidic device consists of two components: an acrylic microwell deposited with an oxygen-sensitive luminescent layer for oxygen (O_2_) detection and a microfluidic module with pneumatically driven acrylic lids to controllably seal the microwells. We successfully measured the metabolic profiles of a single *C. elegans* at key growth and aging stages, determining the following fundamental parameters: basal OCR, ATP-linked OCR, maximal OCR, spare respiratory capacity, OCR due to proton leak, and non-mitochondrial OCR. The bioenergetic health index (BHI), a single value that can represent the bioenergetic health, was calculated from these fundamental parameters to assess the bioenergetic health of a single developing *C. elegans* from the postembryonic development to aging stages.

## 2. Materials and Methods

### 2.1. Principle of Operation

**Figure 1:**
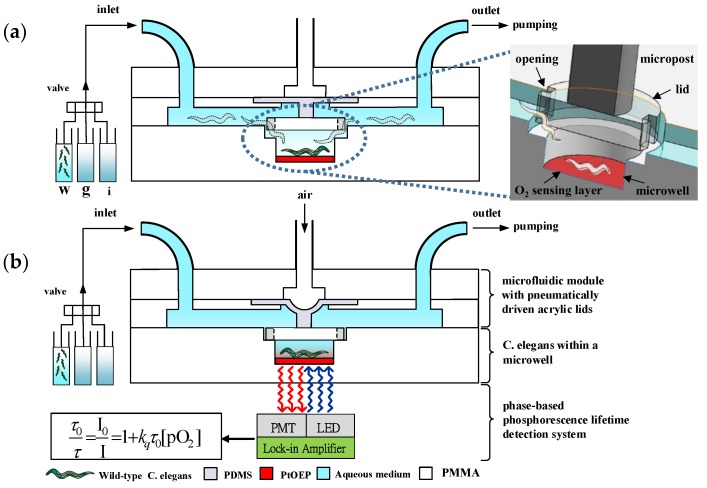
A schematic of a microfluidic device integrated with an optical detection system to measure the oxygen consumption rate (OCR) of a single developing *Caenorhabditis elegans* (*C. elegans)* from the postembryonic development to aging stages in real time via phase-based phosphorescence lifetime measurement. The device consists of two components: an acrylic microwell deposited with an O_2_ sensing layer and a microfluidic module with a pneumatically driven acrylic lid to controllably seal the microwell. A single *C. elegans* in a centrifuge tube (w), the aqueous solution of growth medium in a centrifuge tube (g), or specific metabolic inhibitors in a centrifuge tube (i) was introduced through the inlet of the microfluidic module at a specific point in time via a three-way valve. (**a**) The single *C. elegans* flowed through and crawled along the microchannel toward the microwell. The rounded acrylic lid with two openings as the access points of the microwell allowed the *C. elegans* to pass and fall into the microwell (inset image in (**a**)). (**b**) The long-term OCR measurement via phase-based phosphorescence lifetime measurement was repeated over time using a periodic three-step operation of refilling the microwell with fresh medium, sealing the microwell, and measuring the oxygen concentration by lowering/raising the acrylic lids. PDMS: polydimethylsiloxane; PMMA: poly(methyl methacrylate; PMT: photomultiplier tube.

**Figure 2:**
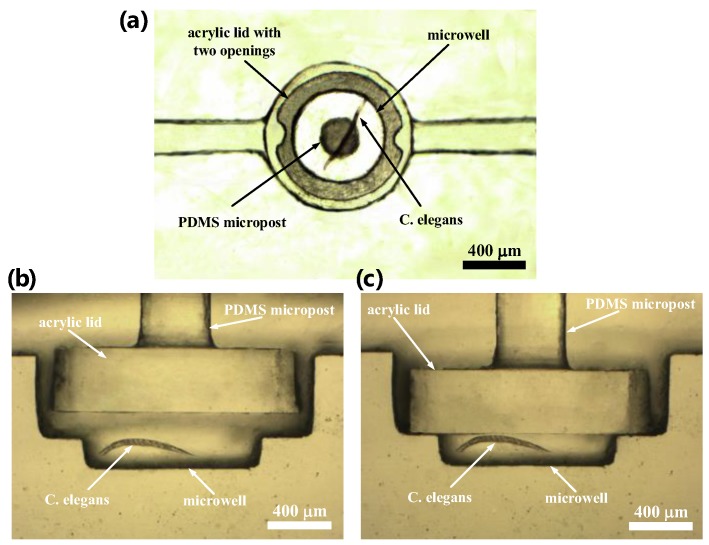
(**a**) A bottom view image of a single adult *C. elegans* entrapped in a microwell and an acrylic lid with two openings set above the microwell. (**b**,**c**) Side view images of a pneumatically driven acrylic lid above the microwell to controllably open and seal the microwell.

A microfluidic device integrated with an optical detection system was developed to measure the OCR of a single developing *C. elegans* via phase-based phosphorescence lifetime detection (Figure 1). The microfluidic device consists of two components: an acrylic microwell deposited with Pt(II) octaethylporphyrin (PtOEP, *λ_ex_* = 381 nm, *λ_em_* = 646 nm, Sigma Aldrich, St. Louis, MO, USA) as an oxygen-sensitive luminescent layer for O_2_ detection [18] and a microfluidic module with a pneumatically driven acrylic lid to controllably seal the microwell. PtOEP displays strong room-temperature phosphorescence with a long lifetime and does not consume oxygen or generate toxic byproducts in the sensing process [18]. Measuring the luminescent phosphorescence lifetime to quantify O_2_ concentrations has been shown to have a high sensitivity and stability. The luminescence lifetime is an intrinsic property of the oxygen-sensitive luminescent dye and is therefore insusceptible to the intensity variation of the incident light or the inhomogeneous in the thickness/distribution of an oxygen-sensitive layer [19,20,21,22]. The acrylic microwell and lid were chosen due to their low oxygen diffusivity to increase the sensor stability and sensitivity. A single *C. elegans* at a preset developmental stage (Table 1) was introduced into the microfluidic device using a micro-diaphragm pump (T5-1IC-03-1EEP, Parker Hannifin Co., Cleveland, OH, USA) at a low flow rate. The single *C. elegans* flowed through and crawled along the microchannel toward the microwell, where a pneumatically driven acrylic lid (1.2 mm in diameter) was closely placed above the microwell. The rounded acrylic lid was fabricated to have two openings as the access points of the microwell, which allowed the *C. elegans* to pass and fall into the microwell (inset image in Figure 1a). The position of the lid close above the microwell effectively prevented the trapped *C. elegans* from creeping or flowing out of the microwell. An air-pressure system, described in our previous work [23], was pneumatically driven to press an acrylic lid attached to the end of a piston to seal the microwell (0.8 mm in diameter, 0.2 mm deep) for OCR measurements (Figure 1b). Figure 2 shows close-up images of a single adult *C. elegans* entrapped in a microwell and a pneumatically driven acrylic lid above the microwell to controllably seal the microwell. The acrylic lid prohibited oxygen diffusion through the microwell that contained a single *C. elegans*. The isolation of a single *C. elegans* in a temporarily sealed microwell, especially a *C. elegans* at an early developmental or aged stage with less oxygen consumption, enables the amplification of oxygen changes during an O_2_ measurement.

The OCR of a single *C. elegans* inside a temporarily sealed microwell was measured by recording the dissolved oxygen concentration (O_2_) of the medium over time via phase-based phosphorescence lifetime detection (Figure 1b). The modulated ultraviolet light-emitting diode (UV LED) light was directed toward the microfluidic device to excite the oxygen-sensitive luminescent layer (PtOEP), and the light intensity of the corresponding phosphorescence was simultaneously recorded by a photomultiplier tube (PMT). The details of the instruments for measuring the phosphorescence signal to calculate the phase shift (*θ*) are described in Section 2.3. The phosphorescence lifetime (*τ*) was calculated by measuring the phase shift (*θ*) between the modulated UV light and the detected phosphorescence signal. The phase shift (*θ*) changes as the oxygen concentration changing and is related to the phosphorescence lifetime (*τ*) as Equation (1):

(1) tan(θ)=2πfτ,

where *f* is the modulation frequency of the UV LED light. The relation between the luminescence intensity (*I*) and lifetime (*τ*) in the absence (*I*_0_, *τ*_0_) and presence (*I*, *τ*) of oxygen follow the Stern-Volmer equation as follows:

(2) $$\frac{I_{0}}{I}=\frac{\tau_{0}}{\tau }=1+{Κ}_{SV}\left[O_{2}\right],$$

where ${Κ}_{SV}$ is the Stern-Volmer constant, and [O_2_] is the oxygen concentration in the solution. The OCR of a single *C. elegans* in a sealed microwell was then calculated by −d[O_2_]/dt from the measured time-dependent [O_2_] data.

After oxygen measurement, the lid was raised to open the microwell within 1 s and refill the microwell with fresh medium. To enable persistent and long-term OCR measurements, each OCR measurement of a single *C. elegans* inside a microwell was operated by periodically repeating the three-step operation of refilling the microwell with fresh medium, sealing the microwell, and measuring the oxygen concentration. At end of the experiments, the single *C. elegans* was flushed out of the microwell by using high flow rates and raising the lid well above the microwell via negative pressure. For the examination of the metabolic profiles of a single *C. elegans*, aqueous solutions containing specific metabolic inhibitors to block bioenergetic pathways were sequentially introduced through the inlet of the microfluidic module at preset developmental stages to treat a single *C. elegans* inside a microwell. The details of the procedures for measuring metabolic profiles to obtain the fundamental parameters of ATP-linked OCR, reserve respiratory capacity, proton leak, and mitochondrial OCR to calculate the BHI are described in Section 3.2. We measured the metabolic profiles of a single *C. elegans* from postembryonic development (larval stages) to aged adulthood. 

### 2.2. Fabrication of the Microfluidic Device

**Figure 3:**
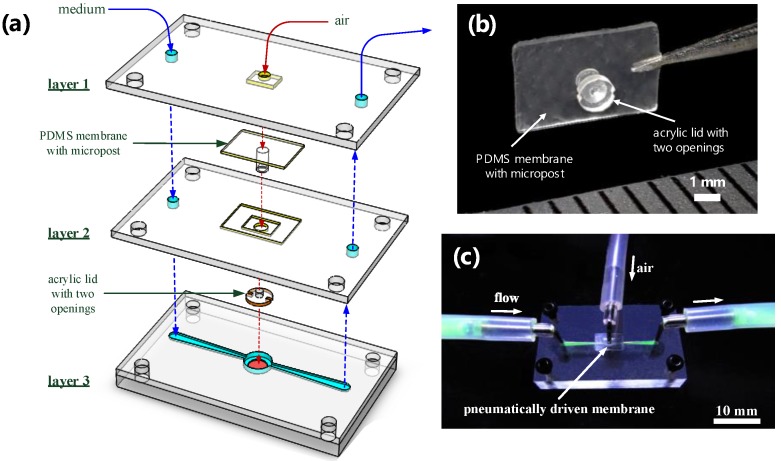
(**a**) An exploded drawing of the microfluidic device, which consists of the acrylic substrate with a microwell containing an oxygen-sensitive luminescent layer (layer 3) and a microfluidic module (layers 1 and 2) with a pneumatically driven acrylic lid above the microwell. (**b**) A close-up of the PDMS membrane with a micropost, where an acrylic lid with two openings is attached to the end of a PDMS micropost. (**c**) An image of the microfluidic device assembled from the microfluidic module and the acrylic substrate, using four screws for ease operation.

Figure 3 shows an exploded drawing and images of the microfluidic device, which consists of an acrylic substrate with a microwell containing an oxygen-sensitive luminescent layer (layer 3) and a microfluidic module (layers 1 and 2) with a pneumatically driven acrylic lid above the microwell. The microfluidic device was assembled by using the top two layers of acrylic structures [poly(methyl methacrylate) (PMMA)] to serve as a microfluidic module, an acrylic lid attached to the end of a polydimethylsiloxane (PDMS, Sylgard 184, Dow Corning, Auburn, MI, USA) micropost and membrane, and a bottom layer of the PMMA substrate with a microwell and microfluidic channels (Figure 3a). All of the acrylic structures were fabricated using a CNC Benchtop Milling Machine (MDX-40A, Roland DG, Irvine, CA, USA). Briefly, the PMMA substrate (layer 3) was first milled to form a well of 0.8 mm in diameter and 0.6 mm in depth and was then milled to form a larger well with a 1.6-mm diameter and 0.4 mm depth above the first well, which can lead to the formation of another microwell with a 0.8 mm diameter and 0.2 mm depth (Figure 2). The larger well above the microwell was designed to accommodate an acrylic lid. Microfluidic channels, 0.2 mm in height and width, were fabricated to connect to the microwell on the PMMA substrate. The oxygen-sensitive luminescent layer (PtOEP) was then deposited into the microwell of the PMMA substrate. First, an 8 wt% solution of polystyrene (Sigma Aldrich, St. Louis, MO, USA) dissolved in toluene and containing PtOEP at a concentration of 200 mM was prepared and spin-coated on the PMMA substrate at 800 rpm for 30 s. After drying at room temperature, the PtOEP film on the surface of the PMMA substrate outside the microwell was removed with a scalpel. The PtOEP film thickness was approximately 1 μm. The polydimethylsiloxane (PDMS) membrane structure with a micropost 0.3 mm in diameter was fabricated and cast from the PMMA mold. An acrylic lid with a 1.2-mm diameter and 0.5-mm thickness with two openings as the access points of the microwell were manually adhered to the PDMS micropost using a PDMS prepolymer as glue. Figure 3b shows a close-up view of the PDMS membrane with a micropost, where an acrylic lid with two openings was attached to the end of the PDMS micropost. Finally, we assembled the microfluidic module (layers 1 and 2) containing an actuated acrylic lid with the PMMA substrate (layer 3) by using four screws for ease operation (Figure 3c). The assembly animation of all three layers and components are shown in Video S1 in the Supplementary Materials.

### 2.3. Optical Detection System for Oxygen Consumption Rate Measurement

**Figure 4:**
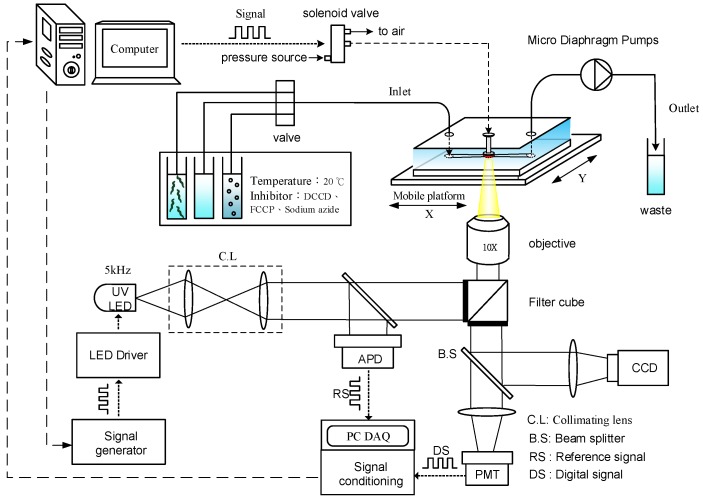
A schematic of the optical detection system, equipped with a UV LED as the light modulation source, to determine the OCR of a single *C. elegans* in real time via phase-based phosphorescence lifetime detection. The phosphorescence lifetime (τ) was calculated by measuring the phase shift (θ) between the modulated reference signal (RS) and the phosphorescence detected signal (DS) by using digital lock-in analysis. An air-pressure system was pneumatically driven to press the acrylic lid to seal the microwell. The aqueous solution of growth medium or specific metabolic inhibitors kept at 20 °C to treat a single *C. elegans* inside a microwell was introduced through the inlet of the microfluidic module at a specific point in time via a three-way valve. (C.L: collimating lens; B.S: beam splitter; APD: amplified photodetector.

Figure 4 shows a schematic of the optical detection system, equipped with a UV LED as the light modulation source, to determine the OCR of a single *C. elegans* in real time via phase-based phosphorescence lifetime detection. The details of the facility setup and data acquisition were described in our previous work [23,24,25]. Briefly, the optical detection system utilized a 3 W high power UV LED (390 nm, Edison Opto Corp., Taipei, Taiwan) as the light modulation source. The UV LED modulated at a frequency of *f* = 5 kHz was used for phase-based phosphorescence lifetime detection because a modulation frequency (*f*) higher than 5 kHz requires high-speed (~10 MHz), expensive data acquisition hardware, which would increase the complexity of the detection system. The modulated excitation light was directed toward the microfluidic device to illuminate the oxygen-sensitive luminescent layer through a collimating lens, a filter unit (Ex: 390 nm; Em: 420 nm; Dm: 400 nm), and an objective lens with 10× magnification. The reference signal (RS) was recorded by measuring the light intensity of the modulated excitation UV light. The detection signal (DS) was recorded by simultaneously measuring the light intensity of the corresponding phosphorescence with a PMT (R928, Hamamatsu, Shizuoka, Japan) in real time along with the RS (the modulated UV light). Both the RS and DS signals were sampled at 50 kHz through the signal conditioning board to produce the averaged data points at a rate of 1 Hz [24]. The phase shift (θ) between the RS and DS was determined by using digital lock-in analysis via a custom-made LabVIEW program (National Instruments, Inc., Austin, TX, USA). An air-pressure system was set up to pneumatically drive an acrylic lid to controllably seal the microwell via three-way solenoid valves (Lee, Inc., Merriam, KS, USA). The aqueous solution of growth medium or specific metabolic inhibitors kept at 20 °C in a thermostatic water bath to treat a single *C. elegans* inside a microwell was introduced through the inlet of the microfluidic module at a specific point in time via a three-way valve.

### 2.4. Stern-Volmer Calibration Curve

The calibrated mixtures with different dissolved oxygen concentrations ranging from 0 to 9.12 mg/L were respectively prepared by bubbling the growth medium (20 °C, pH 5.5) with air or N_2_ gas. The oxygen level of the calibrated mixtures was continuously monitored by Clarke microelectrode sensors (DO-5510, LUTRON, Taipei, Taiwan). The calibrated mixture with continuous bubbling of air was approximately 9.12 mg/L. The calibrated mixtures solutions were then introduced into the device, the glass lid was pushed down to seal the microwell, and calibration tests were performed immediately. Figure S1 shows the variation of the phase shift (*θ*) and the corresponding Stern-Volmer calibration curve of the normalized lifetime (*τ_0_/τ*) as a function of the dissolved oxygen concentration, which was measured by a modulated excitation light at 5 kHz. The luminescence lifetime (*τ_0_*) in the absence of oxygen was 46 μs. In our Stern-Volmer calibration curve, a nearly linear relationship was observed between the normalized lifetime (*τ_0_/τ*) and the dissolved oxygen concentration. The limit of detection of the dissolved oxygen in liquid was 0.02 mg/L, and the response time of PtOEP sensing layers was less than one second [24]. Besides, the pH and CO_2_ level of the aqueous solution has been proven to have no obvious interferences in oxygen measurements using phosphorescent Pt(II) porphyrin-based luminescence oxygen sensors [26,27,28]. 

### 2.5. Worm Culture and Metabolic Inhibitors

Wild-type *C. elegans* N_2_ was obtained from the Caenorhabditis Genetics Center (CGC, University of Minnesota) and cultured as previously described [29]. Briefly, the worms were cultured at 20 °C on standard nematode growth medium agar seeded with *Escherichia coli* (OP50). To synchronize the growth stage of the nematodes, adult worms were treated with NaClO solution until the skin of each individual was mostly destroyed to expose the eggs. The eggs were cultured on fresh nematode growth medium agar plates overnight at 20 °C until hatching. We collected age-synchronous wild-type N_2_ worms hatched at different development stages from the postembryonic development to aged adult stages and then transferred the worms to different microtiter plates for experimentation, as shown in Table 1. We picked a single wild-type N_2_ worm at preset development stages using a picker made from thin platinum wire and then loaded the worm into the microfluidic device with K-medium (50 mM NaCl, 30 mM KCl, 10 mM NaOAc; pH 5.5) for experimentation [30]. In K-medium, worms survived within a pH range of 3.2 to 11.8 for 96 h without significant (*p* > 0.05) lethality [31].

Dicyclohexylcarbodiimide (DCCD), carbonyl cyanide 4-(trifluoromethoxy) phenylhydrazone (FCCP), and sodium azide were purchased from Sigma-Aldrich (St. Louis, MO, USA). The final concentrations of the metabolic inhibitors used in our experiments were prepared according to the data of Luz et al. [5]. Thus, 20 μM DCCD and 25 μM FCCP were used for *C. elegans* at ages of 1.5 to 6 days, whereas 22.5 μM DCCD and 27.5 μM FCCP was for *C. elegans* at ages of 7 to 11 days. The final concentration of sodium azide was approximately 10 mM and 12 mM for 1.5- to 2-day-old and 2.5- to 11-day-old *C. elegans*, respectively. The metabolic inhibitors, which were used at these final concentrations, enabled the production of the maximum change in OCR without inducing death during the experiments. Concentrated stocks of DCCD and FCCP were prepared in dimethyl sulfoxide (DMSO) at 10 mM and 20 mM, respectively.

## 3. Results

### 3.1. The Basal Oxygen Consumption Rate of a Single C. elegans within a Microwell

**Figure 5:**
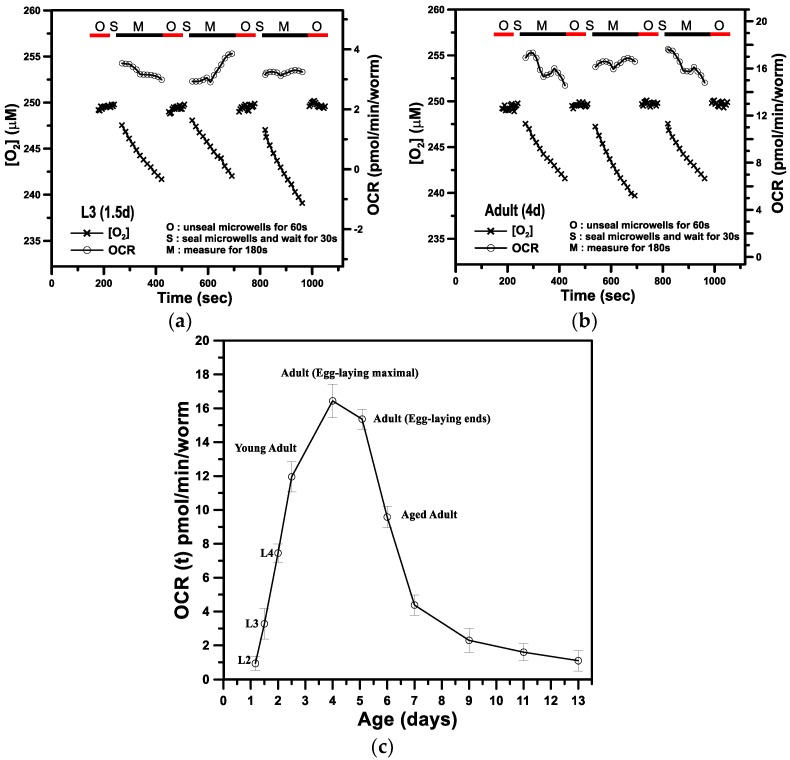
(**a**) The time variation in the aqueous O_2_ concentration ([O_2_], μM) and OCR (−d[O_2_]/dt) for a single *C. elegans* at (**a**) L3 (1.5 days) and (**b**) adult (4 days). (**c**) The basal OCR of a single *C. elegans* inside a sealed microwell from the stages of postembryonic development (L2, L3, and L4) through adulthood to aged adult stages. (Mean ± SEM; *n* = 5).

Figure 5a,b show representative results of the aqueous O_2_ concentration ([O_2_], μM) and OCR (−d [O_2_]/dt, pmol/min/worm) over time for a single *C. elegans* at L3 (1.5-day-old, named 1.5 days) and adult (4-day-old, named 4 days), respectively. Each OCR measurement of a single *C. elegans* inside a microwell was performed via a three-step operation. We first raised the lid to unseal the microwell for 1 min (denoted O-stage) to refill the microwell with fresh K-medium and recover the *C. elegans* to a normal status. In the second stage (denoted S-stage), we lowered the lid to seal the microwell and waited 30 s for stabilization. In the third stage (denoted M-stage), we performed the O_2_ measurement for 3 min to determine the OCR via phase-based phosphorescence lifetime detection. The long-term OCR measurement of a single *C. elegans* was repeated over time by periodically repeating the three-step operation. During the O-stage, the O_2_ concentration was maintained at an approximately constant value of 248 μM over time. During the S- and M-stages, the lid was lowered to create a temporarily sealed microwell, and the O_2_ concentration gradually decreased with time. To calculate transient changes in OCR(t), the time-based differentiation described in our previous work [23,24,25] was used to calculate −d[O_2_]/dt from the measured time-dependent [O_2_] data. The measured OCR(t) represents the basal respiration (also denoted basal OCR), which is the minimal OCR required to maintain basic physiological functions. The mean OCR values of a single developing *C. elegans* for the three successive measurements at 1.5 and 4 days inside a microwell were 3.28 ± 0.84 and 16.44 ± 0.82 pmol/min/worm, respectively. 

Figure 5c shows the basal OCR of a single *C. elegans* as a function of age from postembryonic development (L2 to L4 stages) through adulthood to 13-day-old adults inside a microfluidic device. We performed five separate runs with five different *C. elegans* at each time point to interpret possible individual differences. The basal OCR increased in a linear manner from postembryonic development (L2 to L4 stages), reached the maximum in 4-day-old adults, and gradually decreased to the minimum with 13-day-old adults, which is consistent with previous reports [2]. The basal OCR for a single *C. elegans* at ages younger than L2 (1 day) or for an adult older than 13 days was unmeasurable due to the lower oxygen consumption (extremely low OCR) at these stages, which was beyond the detection limit of our current system. The mean basal OCR at the 13-day-old aged adult stage was as low as 1.1 pmol/min/worm. We attempted to perform the O_2_ measurement by increasing the measuring time from 3 min to 5 or 10 min to determine the OCR for a single *C. elegans* at ages younger than L2 or older than 13 days. However, the increase of the measuring time also increased the photobleaching effect, leading to the significant increase of the signal noises and decrease of the resolution of the proposed sensor. To overcome this problem, we could trap more than two *C. elegans* in a microwell to amply the OCR, use other dye-supporting matrixes for ptOEP instead of polystyrene to improve sensitivity [32], or use other higher sensitive dyes such as Platinum(II) octaethylporphyrin ketone (PtOEPK) [33]. The mean lifespan of wild-type *C. elegans* was about 14–16 days under normal culture conditions. At this dead stage, the basal OCR was close to 0 pmol/min/worm. Moreover, the increase in basal OCR from postembryonic development (L2) until 4-day-old in healthy adults is mostly attributed to the increase in mitochondrial quantity, whereas the decrease in basal OCR afterwards could be due to the decrease in mitochondrial quantity and/or the increased quantity of dysfunctional mitochondria. Interestingly, the maximal basal OCR of 4-day-old adults coincided with the egg-laying maximum of *C. elegans*, which usually occurred in 4-day-old adults and then decreased exponentially. The coincidence of these two maximum is a reasonable result because a great deal of energy would be needed for reproduction [2]. In contrast to the active 4-day-old adults with maximal basal OCR, *C. elegans* adults older than 6 days had a gradual decrease in the basal OCR to the minimum of 1.5 pmol/min/worm for 13-day-old adults. With such a low basal OCR, the *C. elegans* adults older than 9 days until the dead stage were found to be less active with slow or even no motion (Table 1). These results suggest that the aging process coincides with the gradual decrease in the basal OCR, i.e., respiratory activity.

### 3.2. Metabolic Profiles and the Bioenergetic Health Index of a Single Developing C. elegans

**Figure 6:**
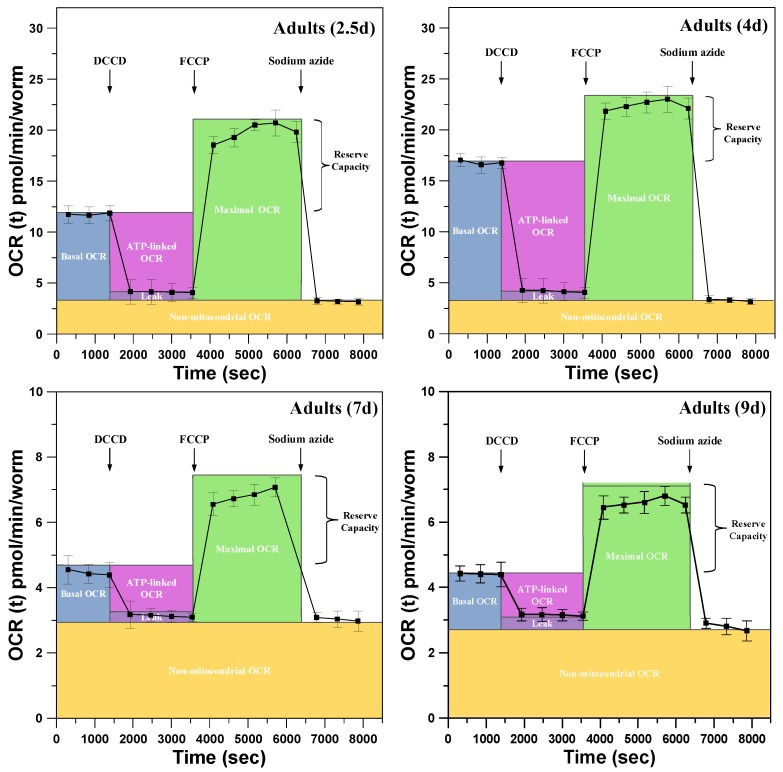
The representative results of the metabolic profiles of a single developing *C. elegans* at ages of 2.5, 4, 7, and 9 days obtained by sequentially adding metabolic inhibitors (DCCD, FCCP, and sodium azide) to block bioenergetic pathways. The metabolic profiles show the following fundamental parameters: basal OCR (blue), ATP-linked OCR (pink), maximal OCR (green), reserve respiratory capacity, OCR due to proton leak (purple), and non-mitochondrial OCR (yellow). (Mean ± SEM; *n* = 3).

For the examination of the metabolic profiles of a single *C. elegans* at key growth and aging stages, aqueous solutions containing specific metabolic inhibitors (DCCD, FCCP, and sodium azide) to block bioenergetic pathways were sequentially introduced through the inlet of the microfluidic module to monitor changes in mitochondrial function. Figure 6 show representative results of the metabolic profiles of a single developing *C. elegans* at ages of 2.5, 4, 7, and 9 days obtained by sequentially adding metabolic inhibitors to block bioenergetic pathways. The metabolic profiles show the following fundamental parameters: basal OCR, ATP-linked OCR, maximal OCR, reserve respiratory capacity, OCR due to proton leak, and non-mitochondrial OCR. At the onset of measurements, the basal OCR was measured through three repeats, where each repeat included the three-step operation of O-stage (60 s)/S-stage (30 s)/M-stage (180 s). At the end of the third repeat, DCCD, an inhibitor of mitochondrial ATP synthase, was introduced to treat a single *C. elegans* to inhibit the activity of ATP synthase, thus blocking the phosphorylation of ADP to ATP. The decrease in basal OCR that is coupled to ATP turnover is denoted as ATP-linked OCR. Note that oligomycin and DCCD are typical ATP synthase inhibitors used for cellular metabolic analysis [34]. However, the bulky compound oligomycin was found to be ineffective at inhibiting ATP synthase, likely due to the limited penetration of the *C. elegans* collagenous cuticle. Instead, DCCD has been proven to be more effective in inhibiting ATP synthase in *C. elegans* at all ages [5]. The inhibition of ATP synthase provides a measure of the amount of oxygen consumption coupled directly to ATP production. The remaining rate of mitochondrial respiration represents the proton leak that results in oxygen consumption without ATP production (OCR due to proton leak). After the inhibition of mitochondrial ATP synthase, FCCP, the proton ionophore, was introduced into the microfluidic device to treat a single *C. elegans*. Immediately upon exposure to FCCP, the OCR increased as the mitochondrial inner membrane became permeable to protons and reached the maximal OCR. The reserve respiratory capacity, which is calculated by subtracting the maximal OCR from the basal OCR, represents the mitochondrial reserve energy available to increase energy production in the face of chronic and acute stress [34]. Finally, upon treatment with sodium azide, which blocks mitochondrial respiration, only the non-mitochondrial OCR can be measured. 

**Figure 7:**
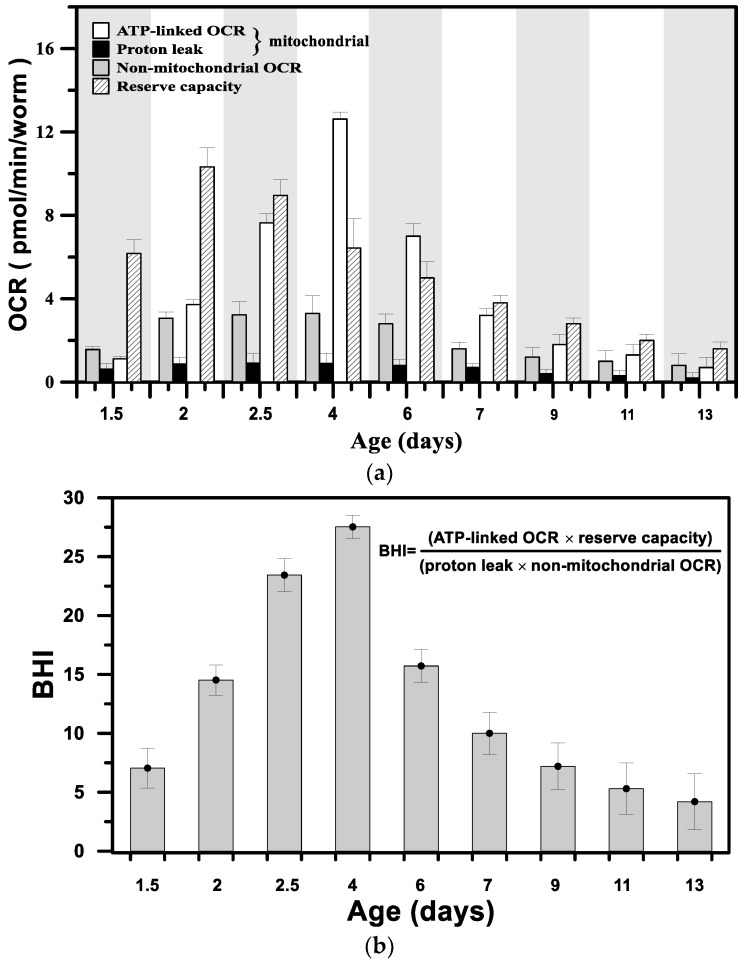
(**a**) The variation in ATP-linked OCR, proton leak, reserve respiratory capacity, and non-mitochondrial OCR in pmol/min/worm as a function of age from the stages of postembryonic development through adulthood to aged adult stages. (**b**) The bioenergetic health index (BHI) as a function of age, which was calculated from the fundamental parameters in (**a**).

Figure 7a shows the variations in ATP-linked OCR, proton leak, reserve respiratory capacity, and non-mitochondrial OCR in pmol/min/worm as a function of age from the postembryonic development through adulthood to aged adult stages. Figure 7b shows the BHI as a function of age, which was calculated from the fundamental parameters in Figure 7a using the following formula [35]:

(3) $$\mathrm{BHI}=\frac{\left(\mathrm{ATP}-\mathrm{linked}\;\mathrm{OCR}\times \mathrm{reserve}\;\mathrm{capacity}\right)}{\left(\mathrm{proton}\;\mathrm{leak}\times \mathrm{non}-\mathrm{mitochondrial}\;\mathrm{OCR}\right)},$$

The BHI, a single value that can represent bioenergetic health, is sensitive to the mitochondrial functionality of a single developing *C. elegans* during the growth and aging stages. Equation (3) captures positive aspects of bioenergetic function (reserve capacity and ATP-linked OCR) relative to potentially deleterious aspects (non-mitochondrial OCR and proton leak). As shown in Figure 7b, the changes in BHI were correlated to *C. elegans* development stage, with the highest BHI = 27.5 in 4-day-old adults, and BHI = 7 and 4.2 at the ages of 1.5 and 13 days, respectively. 

As expected, the variation in the BHI was consistent with that of basal OCR, with the highest values found in 4-day-old adults (Figure 5c). However, the high basal OCR could not exactly reflect the status of mitochondrial functionality; for example, the treatment of normal cardiomyocytes with 4-hydroxynonenal (oxidative stress) to damage the inner mitochondrial membrane, i.e., the loss of the mitochondrial functionality, has been previously reported to significantly increase the basal OCR due to the increase in ATP-linked OCR and proton leak [36]. Instead, the BHI can faithfully reflect both positive and deleterious parameters. The high BHI indicates that the developing *C. elegans* possesses high bioenergetic function with a high reserve capacity, high ATP-linked OCR, and low proton leak from healthy and well-developed mitochondria. For the developing adult *C. elegans* at the age of 4 days, the bioenergy is mostly generated through oxidative phosphorylation (mitochondrial respiration), corresponding to the highest ATP-linked OCR in Figure 7a. For the developing *C. elegans* at the early postembryonic development age (1.5 days, L3 stage), the ATP-linked OCR is low, corresponding to a low BHI, because the bioenergy is mostly generated from glycolysis without oxygen consumption. At this early stage, a massive quantity of new mitochondria are formed in the cell and might not be fully functional [37]. Glycolysis serves as a major bioenergy source during early development and significantly decreases with age [38]. In addition, the reserve capacity was high at these early postembryonic stages (1.5, 2, and 2.5 days; Figure 7a) to provide the capacity to deal with the increased bioenergetic demand and metabolic stress during these stages. For developing *C. elegans* adults older than 4 days, the BHI gradually decreased to reach the lowest BHI = 4.2 at the age of 13 days. The aging process accompanied by gradually increasing unhealthy mitochondria [39] leads to a progressive deterioration in the bioenergetic function of mitochondria, which manifests as the low ATP-linked OCR and low reserve capacity (Figure 7a). The aged *C. elegans* with a low BHI, which possesses insufficient ATP to meet the metabolic demands, shows inactivity with slow or even no motion. These results suggest that the BHI could serve as a dynamic index to show the status of the bioenergetic health of *C. elegans* in growth and aging stages. The change in the mitochondrial functionality correlated with the growth and aging progress of a single developing *C. elegans* still requires extensive and intensive study in conjunction with other biochemical assays to provide additional information, such as the mitochondrial DNA mutations that accompany aging. However, the determination of the possible mechanism of the mitochondrial malfunction in growth and aging progress is beyond the scope of this study. 

## 4. Conclusions

We developed a microfluidic device integrated with an optical detection system to measure the OCR of a single developing *C. elegans* from postembryonic development to aging stages in real time via phase-based phosphorescence lifetime measurement. Our proposed platform demonstrates for the first time the feasibility of measuring the bioenergetic profiles of a single *C. elegans* entrapped in a microwell to assess the BHI from the stages of postembryonic development through adulthood to aging stages. We also demonstrated that the BHI, a single value that is sensitive to mitochondrial functionality, could serve as a dynamic index to show the status of the bioenergetic health of *C. elegans* during the growth and aging processes. The microfluidic device can easily be extended from the current proof of concept into multiple microwells for a high-throughput measurement and can be integrated with other microfluidic components for the on-demand sorting, isolation, and manipulation of *C. elegans*. We are continuing to use our proposed microfluidic device for assessing bioenergetic dysfunction using the BHI as a dynamic index for *C. elegans* exposed to environmental toxins, therapeutic agents or oxidative stress. Our proposed platform provides potential for studies of bioenergetic metabolism of a developing organism (*C. elegans*) for a wide variety of biomedical applications that relate to mitochondrial malfunction and diseases.

## Figures and Tables

**Table 1:** Developmental stages of wild-type N_2_ worms used for experimentation.

Stage	Feature	Mean Age (day)	OCR (pmol/min/worm)
L1	558 somatic nuclei	0.5	NA
L2	germ cell divisions continue	1.2	0.94 ± 0.41
L3	forms spermathecal/uterus	1.5	3.28 ± 0.89
L4	generate oocytes	2	7.45 ± 0.55
Young adults	959 somatic nuclei	2.5	11.96 ± 0.91
Adults	egg-laying maximum	4	16.44 ± 0.96
Adults	egg-laying ends	5	15.36 ± 0.59
Aged adults	slow motion	6	9.58 ± 0.64
Aged adults	slow motion	7	4.38 ± 0.41
Aged adults	less motion	9	2.33 ± 0.71
Aged adults	less motion	11	1.65 ± 0.52
Aged adults	no motion	13	1.12 ± 0.62

## Supplementary Materials

The following are available online at http://www.mdpi.com/1424-8220/18/8/2453/s1, Figure S1: The variation of the phase shift (θ) and the corresponding Stern–Volmer calibration curve of the normalized lifetime (τ_0_/τ) as a function of the dissolved oxygen concentration, which was measured by a modulated excitation light at 5 kHz., Video S1: The assembly animation of all three layers and components.

## References

[1] Wilson D.F., Harrison D.K., Vinogradov S.A. (2012). Oxygen, ph, and mitochondrial oxidative phosphorylation. J. Appl. Physiol..

[2] Suda H., Shouyama T., Yasuda K., Ishii N. (2005). Direct measurement of oxygen consumption rate on the nematode caenorhabditis elegans by using an optical technique. Biochem. Biophys. Res. Commun..

[3] Stackley K.D., Beeson C.C., Rahn J.J., Chan S.S.L. (2011). Bioenergetic profiling of zebrafish embryonic development. PLoS ONE.

[4] Huang S.-H., Huang K.-S., Yu C.-H., Gong H.-Y. (2013). Metabolic profile analysis of a single developing zebrafish embryo via monitoring of oxygen consumption rates within a microfluidic device. Biomicrofluidics.

[5] Luz A.L., Rooney J.P., Kubik L.L., Gonzalez C.P., Song D.H., Meyer J.N. (2015). Mitochondrial morphology and fundamental parameters of the mitochondrial respiratory chain are altered in caenorhabditis elegans strains deficient in mitochondrial dynamics and homeostasis processes. PLoS ONE.

[6] Koopman M., Michels H., Dancy B.M., Kamble R., Mouchiroud L., Auwerx J., Nollen E.A.A., Houtkooper R.H. (2016). A screening-based platform for the assessment of cellular respiration in caenorhabditis elegans. Nat. Protoc..

[7] Payne B.A.I., Chinnery P.F. (2015). Mitochondrial dysfunction in aging: Much progress but many unresolved questions. BBA Bioenerg..

[8] McBride H.M., Neuspiel M., Wasiak S. (2006). Mitochondria: More than just a powerhouse. Curr. Biol..

[9] Felix M.A., Braendle C. (2010). The natural history of caenorhabditis elegans. Curr. Biol..

[10] Aubry G., Lu H. (2014). A perspective on optical developments in microfluidic platforms for caenorhabditis elegans research. Biomicrofluidics.

[11] Shanmugam M.M., Santra T.S. (2016). Microfluidic devices in advanced caenorhabditis elegans research. Molecules.

[12] Gupta B.P., Rezai P. (2016). Microfluidic approaches for manipulating, imaging, and screening c. Elegans. Micromachines.

[13] Yang J.P., Chen Z.G., Ching P.Y., Shi Q.J., Li X.C. (2013). An integrated microfluidic platform for evaluating in vivo antimicrobial activity of natural compounds using a whole-animal infection model. Lab Chip.

[14] Crane M.M., Chung K., Stirman J., Lu H. (2010). Microfluidics-enabled phenotyping, imaging, and screening of multicellular organisms. Lab Chip.

[15] Song P.F., Zhang W.Z., Sobolevski A., Bernard K., Hekimi S., Liu X.Y. (2015). A microfluidic device for efficient chemical testing using caenorhabditis elegans. Biomed. Microdevices.

[16] Zhang B.B., Li Y.B., He Q.D., Qin J., Yu Y.Y., Li X.C., Zhang L., Yao M.C., Liu J.S., Chen Z.G. (2014). Microfluidic platform integrated with worm-counting setup for assessing manganese toxicity. Biomicrofluidics.

[17] Lin Y.W., Huang S.H. Metabolic profile analysis of a single caenorhabditis elegans across the lifespan within a microfluidic device. Proceedings of the 2017 19th International Conference on Solid-State Sensors, Actuators and Microsystems (TRANSDUCERS).

[18] Ji S., Wu W., Wu Y., Zhao T., Zhou F., Yang Y., Zhang X., Liang X., Wu W., Chi L. (2009). Real-time monitoring of luminescent lifetime changes of ptoep oxygen sensing film with led/photodiode-based time-domain lifetime device. Analyst.

[19] Gruber P., Marques M.P.C., Szita N., Mayr T. (2017). Integration and application of optical chemical sensors in microbioreactors. Lab Chip.

[20] Jahn K., Buschmann V., Hille C. (2015). Simultaneous fluorescence and phosphorescence lifetime imaging microscopy in living cells. Sci. Rep..

[21] Yu Y.C., Kwon M.S., Jung J., Zeng Y.Y., Kim M., Chung K., Gierschner J., Youk J.H., Borisov S.M., Kim J. (2017). Room-temperature-phosphorescence-based dissolved oxygen detection by core-shell polymer nanoparticles containing metal-free organic phosphors. Angew. Chem. Int. Ed..

[22] Li Z., Bassett W.P., Askim J.R., Suslick K.S. (2015). Differentiation among peroxide explosives with an optoelectronic nose. Chem. Commun..

[23] Huang S.-H., Hsu Y.-H., Wu C.-W., Wu C.-J. (2012). Light-addressable measurements of cellular oxygen consumption rates in microwell arrays based on phase-based phosphorescence lifetime detection. Biomicrofluidics.

[24] Huang S.-H., Tsai C.-H., Wu C.-W., Wu C.-J. (2011). Light-directed, spatially addressable oxygen detection in a hydrogel microarray based on phase-based lifetime detection using a digital micromirror device. Sens. Actuators A Phys..

[25] Huang S.H., Huang K.S., Liou Y.M. (2017). Simultaneous monitoring of oxygen consumption and acidification rates of a single zebrafish embryo during embryonic development within a microfluidic device. Microfluid Nanofluid.

[26] Meier R.J., Schreml S., Wang X.D., Landthaler M., Babilas P., Wolfbeis O.S. (2011). Simultaneous photographing of oxygen and ph in vivo using sensor films. Angew. Chem. Int. Ed..

[27] Xu W., Lu S.S., Chen Y.Y., Zhao T.T., Jiang Y.Q., Wang Y.R., Chen X. (2015). Simultaneous color sensing of o-2 and ph using a smartphone. Sens. Actuators B Chem..

[28] Chu C.S., Syu J.J. (2017). Optical sensor for dual sensing of oxygen and carbon dioxide based on sensing films coated on filter paper. Appl. Opt..

[29] Brenner S. (1974). Genetics of caenorhabditis-elegans. Genetics.

[30] Zhu G.L., Yin F.C., Wang L., Wei W.B., Jiang L., Qin J.H. (2016). Modeling type 2 diabetes-like hyperglycemia in c-elegans on a microdevice. Integr. Biol..

[31] Khanna N., Cressman C.P., Tatara C.P., Williams P.L. (1997). Tolerance of the nematode caenorhabditis elegans to ph, salinity, and hardness in aquatic media. Arch. Environ. Contam. Toxicol..

[32] Zhang K., Zhang H.L., Wang Y., Tian Y.Q., Zhao J.P., Li Y. (2017). High sensitivity and accuracy dissolved oxygen (do) detection by using ptoep/poly(mma-co-tfema) sensing film. Spectrochim. Acta A.

[33] Nock V., Blaikie R.J., David T. (2008). Patterning, integration and characterisation of polymer optical oxygen sensors for microfluidic devices. Lab Chip.

[34] Hill B.G., Benavides G.A., Lancaster J.R., Ballinger S., Dell’Italia L., Jianhua Z., Darley-Usmar V.M. (2012). Integration of cellular bioenergetics with mitochondrial quality control and autophagy. Biol. Chem..

[35] Chacko B.K., Kramer P.A., Ravi S., Benavides G.A., Mitchell T., Dranka B.P., Ferrick D., Singal A.K., Ballinger S.W., Bailey S.M. (2014). The bioenergetic health index: A new concept in mitochondrial translational research. Clin. Sci. (Lond.).

[36] Chacko B.K., Zhi D., Darley-Usmar V.M., Mitchell T. (2016). The bioenergetic health index is a sensitive measure of oxidative stress in human monocytes. Redox Biol..

[37] Beeson C.C., Beeson G.C., Schnellmann R.G. (2010). A high-throughput respirometric assay for mitochondrial biogenesis and toxicity. Anal. Biochem..

[38] Facucho-Oliveira J.M., St. John J.C. (2009). The relationship between pluripotency and mitochondrial DNA proliferation during early embryo development and embryonic stem cell differentiation. Stem Cell Rev. Rep..

[39] Bratic A., Larsson N.G. (2013). The role of mitochondria in aging. J. Clin. Investig..

