# Supplementary material for: Bioenergetic Health Assessment of a Single Caenorhabditis elegans from Postembryonic Development to Aging Stages via Monitoring Changes in the Oxygen Consumption Rate within a Microfluidic Device
Source: Sensors (Basel). 2018 Jul 28;18(8):2453. doi: 10.3390/s18082453 (PMC6111518; doi:10.3390/s18082453)
Supplement: Supplementary file 1 [file sensors-18-02453-s001.zip › Sensors-C elegans-Supplement.docx]

Supplementary Information

Bioenergetic health assessment of a single Caenorhabditis elegans from postembryonic development to aging stages via monitoring changes in the oxygen consumption rate within a microfluidic device

Shih-Hao Huang * and Yu-Wei Lin

Department of Mechanical and Mechatronic Engineering, National Taiwan Ocean University, Keelung, 202-24, Taiwan; shihhao@mail.ntou.edu.tw (S.H.); n490524@yahoo.com.tw (Y.W.)

***** Correspondence: shihhao@mail.ntou.edu.tw; Tel.: 886-2-24622192 ext. 3209

Received: date; Accepted: date; Published: date

**Figure S1.** The variation of the phase shift (θ) and the corresponding Stern–Volmer calibration curve of the normalized lifetime (τ_0_/τ) as a function of the dissolved oxygen concentration, which was measured by a modulated excitation light at 5 kHz.
